# Effects of hydrogen peroxide priming on yield, photosynthetic capacity and chlorophyll fluorescence of waterlogged summer maize

**DOI:** 10.3389/fpls.2022.1042920

**Published:** 2022-10-21

**Authors:** Shouran Wang, Juan Hu, Baizhao Ren, Peng Liu, Bin Zhao, Jiwang Zhang

**Affiliations:** State Key Laboratory of Crop Biology and College of Agronomy, Shandong Agricultural University, Tai’an, Shandong, China

**Keywords:** summer maize, H2O2 priming, photosynthesis characteristics, waterlogging stress, grain yield

## Abstract

Extreme rainfall events during the summer maize growth and development periods, which have induced losses in summer maize production. There was a completely randomized block experiment being designed with four treatments: waterlogging for 6 days at the V3 stage (C-W), H_2_O_2_-priming + non-waterlogging (H-CK), H_2_O_2_-priming + waterlogging for 6 days at the V3 stage (H-W) and control (C-CK). This study investigated the effects of H_2_O_2_ priming on yield and photosynthetic parameters of (*Zea mays*. L) summer maize hybrid DengHai605 (DH605) by measuring the leaf area index (LAI), soil and plant analyzer development (SPAD) value, stomatal morphology, gas exchange parameters, and chlorophyll fluorescence parameters. The results showed that the net photosynthetic rate (Pn) was decreased after waterlogging through the stomatal limitation of CO_2_ supply and reduction of PSII photochemical efficiency, which led to the decrease in dry matter accumulation and grain yield. H_2_O_2_ priming increased the number of opening stomas, the stomatal length, and width, thus increasing Ci by 12.1%, which enhanced the Pn by 37.5%. Additionally, H_2_O_2_ priming could improve the energy of dark reaction carbohydrates by increasing the light energy absorption and utilization, alleviating the function of PSII reaction centers, protecting the PSII receptor and donor side, and the electron transport chain. The φEo, φPo, φRo, and Ψo of H-W were increased by 89.9%, 16.2%, 55.4%, and 63.9% respectively, and the φDo was decreased by 23.5%, compared with C-W. Therefore, H_2_O_2_ priming significantly enhanced the PSII photochemical efficiency, and increased the CO_2_ supply in dark reactions to promote carbon assimilation, alleviating the waterlogging-induced damage to maize plant growth and grain yield.

## 1 Introduction

In recent years, due to anthropogenic contributions including industrialization and urbanization, the environmental conditions have deteriorated, causing waterlogging disasters to occur frequently all over the world (Pan, 2022; Voesenek and Bailey-Serres, 2015). Waterlogging, as one of the main abiotic stresses for the normal growth and development of summer maize, seriously harmed crop production in nearly 16% of the world’s agricultural production areas (Seng et al., 2012; Zhou et al., 2020). In the Huang huai hai region, the seedling stage (especially in the V3 stage) of summer maize coincided with the period of frequent rainfall (Yu, 2015). The Huang huai hai Plain was the main summer maize producing area in China, whose planting area and yield accounted for more than 1/3 of all maize planting areas in China (Gao, 2018), and 70% of annual precipitation was concentrated during the growing period of summer maize. Such a large amount of precipitation would lead to frequent rainfall-induced waterlogging, which could seriously affect the yield and quality of summer maize (Ren et al., 2015). It is thus desirable to explore effective management to mitigate the waterlogging damages to maize production.

When water content exceeded 80% of the maximum field water capacity, plant growth and development of summer maize were significantly inhibited, and the greatest impact occurred at the V3 stage (Ren et al., 2017). From the point of view of phenotypic characteristics: The leaves turn yellow and lose their green colour, which decreased the leaf area index and SPAD values, and reduced the production capacity by optical contraction (Ren et al., 2015). From the physiological point of view: Waterlogging stress resulted in stomatal closure, thus blocking the CO_2_ uptake, which significantly reduced the photosynthetic rate of summer maize, and inhibited the ability of photosynthetic assimilation ability (Ren et al., 2018). With the prolonged waterlogging duration, the PSII system of summer maize was damaged, photosynthetic electron transport was blocked, and carbon assimilation was inhibited (Hu et al., 2020; Ren et al., 2020; Yu et al., 2021). So, when the plants encountered waterlogging stress, the stomatal performance and the PSII photoinhibition of leaves were limited, which further reduced production capacity and reduced the grain yield finally.

Chemical priming could improve the plant plasticity to abiotic stresses (Kaori et al., 2020). Plant priming was related to stress “memory” which as a potential way of improving cross-tolerance (When the plants were exposed to a mild stress, the subsequence severe stresses were improved) (Munné-Bosch and Alegre, 2013). H_2_O_2_, as one of the chemical priming agents, played an important role in the stress signal transduction pathway that was essential for crop growth and adaptation to adversity (Gao et al., 2010; Hossain et al., 2015). H_2_O_2_ played a wide role in plants responding to abiotic stresses by regulating a multitude of physiological processes such as acquiring resistance, antioxidant defense, photosynthesis, and stomatal opening (Petrov and Breusegem, 2012; Gdsa et al., 2020). Previous studies have demonstrated that pretreatments with 70 mM H_2_O_2_ favored the increase in net physiological rate, PSII efficiency, plant biomass, and antioxidant activity of leaves in soybean (*Glycine max* [L.] Merr.) (Andrade et al., 2018). It has been well summarized in several reviews that H_2_O_2_ priming could enhance plant tolerance to abiotic stress (Perez and Brown, 2014; Ayano et al., 2015; Kaori et al., 2020). Recent publications have reported that exogenous treatment of H_2_O_2_ could preserve plants from low temperature (Wahid et al., 2007), drought (Zhang et al., 2022), salt (Gondim et al., 2009), waterlogging (Andrade et al., 2018), and other stresses. Nonetheless, the use of H_2_O_2_ as a chemical priming agent has been rarely studied under waterlogging stress, especially in H_2_O_2_ alleviating the effects of waterlogging stress on photosynthetic characteristics (Perez & Brown, 2014; Ayano et al., 2015). As one of the most basic physiological activities of plants, photosynthesis which provided material and energy for plant life activities was also one of the important indicators of the ability of plants to withstand adversity and stress. The photosynthesis in the leaves decreased under waterlogging pressure, which would reduce crop yield and quality (Ashraf and Harris, 2013; Tian et al., 2019). This study was to investigate the effects of H_2_O_2_ priming on the yield and photosynthetic characteristics of summer maize subjected to waterlogging stress, thus providing theoretical and technical support for the chemical regulation of summer maize under waterlogging stress.

## 2 Materials and methods

### 2.1 Experimental materials and design

This experiment was conducted in 2020 and 2021 to explore the effects of H_2_O_2_ priming on waterlogged summer maize at the Shandong Agricultural University experimental farm (36.10°N, 117.09°E). Summer maize hybrid Denghai605 (DH605) was used as experimental materials. DH605 was approved by the approval committee of China in 2010 which was suitable for planting with in Shandong province and widely grown in China (Hu et al., 2020). Seed soaking for 12 h with 0.1% H_2_O_2_ [relevant literature showed that 0.1% H_2_O_2_ significantly promoted the germination of maize seeds (Zhang, 1996)]. Summer maize was sown on June 9 in 2020 and 2021 with the density of 67500 plants ha^-1^, and waterlogging at the V3 stage for 6 days (Extreme rainfall events were frequent in the Huang huai hai region from June to July which was the V3 stage of summer maize (**Supplementary Figure 1**). At the same time, the plant growth and development of summer maize were significantly inhibited after waterlogging, and the greatest impact occurred at the V3 stage (Ren et al., 2017)). For details, see (**Table 1**). N, P, and K fertilizers were applied as base fertilizers: 210 kg ha^-1^ N (urea, 46% N), 52.5 kg ha^-1^ P_2_O_5_ (calcium superphosphate, 17% P_2_O_5_), and 67.5 kg ha^-1^ K_2_O (muriate of potash, 60% K_2_O). Management of other diseases, insects, grasses, and pests by reference to high-yield fields. The details were shown in (Hu et al., 2021). Complete randomized block design. Plot planting test was used in this experiment. A square plot was formed by 4 pieces of PVC board with 4 m × 4 m to form a 16 m^2^ reservoir. Each piece of PVC plate was a length 4 m and width 2.3 m, which was used to bury 2.0 m below the underground and 0.3m above ground to facilitate the formation of a 2-3 cm water layer. The climatic conditions of the maize growing season in 2020 and 2021 are shown in (**Supplementary Figure 1**). The total annual precipitations were 805.8 and 939.1mm in 2020 and 2021, and mean temperatures were 25.1 and 25.3°C during the summer maize growth cycles in the two years.

**Table 1:** Waterlogging treatments for summer maize in the field from 2020 to 2021.

Abbreviation	Treatment
C-CK	Non-waterlogging treatment
C-W	Waterlogging at V3 stage
H-CK	H_2_O_2_ priming – Non-waterlogging treatment
H-W	H_2_O_2_ priming - Waterlogging at V3 stage

### 2.2 Leaf area index

After 6 days of continuous waterlogging, six representative plant samples were signed from each plot (H-CK, H-W, C-CK, C-W) to measure and calculate leaf area. LAI was calculated as follows (Ren et al., 2016).

Leaf area (cm^2^)=L (cm)×W (cm)×0.75

LAI=(leaf area per plant×plant number per plot)/plot area

### 2.3 Soil and plant analyzer development value

After 6 days of continuous waterlogging, a chlorophyll meter (SPAD-502, Soil-plant Analysis Development Section, MinoltaMinolta-CameraCo., Osaka, Japan) was used to measure 12 times of each plot to obtain the leaf SPAD value.

### 2.4 Dry matter accumulation

After 6 days of continuous waterlogging, three typical plant samples were selected from each plot. The samples were placed in the oven at 105°C in a force-draft oven for 30 min, and then dried to constant weight at 80°C and weighed separately. Refer to the specific method as follows (Ren et al., 2017).

### 2.5 Stomatal phenotypic characteristics of leaves

After 6 days of continuous waterlogging, the stomatal samples were collected during 9:00-11:00 am. First of all, we collected stomatal imprints from the middle section of the new fully expanded leaf using colorless transparent nail polish. Three leaves were selected for each plot and two replicates per leaf. After drying, the leaf was cut with scissors and then pasted with colorless transparent tape on the leaf with nail polish to be tested on the slide (Zheng et al., 2013; Yan et al., 2021). The imprints were observed and photographed in the laboratory with a microscope (Biological microscope, Nikon of Japan, Ni-u). A total of 30 photos (3 × 2 × 5) were taken from 5 randomly selected fields of vision at 200 × for stomatal density (SD, number of pores per unit area) and proportion of open stomata (POS), and 30 photos were taken at 400 × for open stomatal length (SL), width (SW), perimeter (SP) and area (SA) measurement and recording (Li et al., 2018).

### 2.6 Leaf gas exchange parameters

After 6 days of continuous waterlogging, the leaf gas exchange parameters: the net photosynthetic rate (Pn), stomatal conductance (Gs), intercellular CO_2_ concentration (Ci), and transpiration rate (Tr) of ear leaves were measured between 10:00-12:00 am under saturating irradiance using a portable infrared gas analyzer (CIRAS-3, PP System, Hanstaech, U.K) and then calculated the pore limit value (Ls). Leaves with consistent growth and good light exposure under different treatments were selected. Five leaves were measured randomly for each treatment. Measurement conditions were kept consistent: LED light source and photosynthetically available radiation (PAR) of 1600 μmol m^-2^. CO_2_ concentration was maintained at a constant level of 360 μmol mol-1 using a CO_2_ injector with a high-pressure liquid CO_2_ cartridge.

Ls=(Ca-Ci)/Ca, in this formula, Ci was intercellular CO_2_ concentration and Ca was environmental CO_2_ concentration (Berry, 1982; Seng et al., 2012).

### 2.7 Chlorophyll fluorescence parameters

After 6 days of continuous waterlogging, photo-induced transients’ photoinduced transients (**Supplementary Table 1**) of Fo, Fm, Fv/Fm, Wk, Vj, TRo/CSm, ETo/CSm, DIo/CSm, ABS/CSm, ψO, φDo, φEo, φPo, PI_ABS_ of the five typical leaves each plot was measured by M-PEA (Multi-Function Plant Efficiency Analyser, Hansatech Instruments Limited, U.K). See (Strasser, 2004) for the specific meanings of the parameters. The M-PEA data version was 1.10, the measurement duration was 10.0s, the number of data points was 145, and the red-light intensity was 3000 μmol m^-2^ s^-1^. The determination time was consistent with that of photosynthetic parameters. The leaves should be dark-treated with clamps for 20 min before determination. Representative plants with good growth should be selected, with 5 plants in each treatment.

### 2.8 Grain yield

At R6, 30 ears harvested continuously selected from three rows were used to determine yield and ear traits. Grain yield was calculated as follows (Ren et al., 2016).

Grain yield (kg ha^-1^) = Harvest ear (ears ha^-1^)×Number of kernel per ear×1,000-grain weight (g/1000 grains)/10^6^×(1-moisture content%)/(1-14%)

### 2.9 Statistical analysis

Microsoft Excel 2016 (Microsoft, Redmond, WA, USA) was used for data processing and analysis. IBM SPSS Statistics 18.0 (IBM Cor-poration, Armonk, NY, USA) was used for data analysis. Analysis of variance (ANOVA) and LSD (Least significant difference) tests was used for comparisons analysis. The coefficient of pearson was used for the analysis of correlation. The significant differences are at P<0.05. The data were plotted using Origin 2021 (OriginLab Corporation, MA, USA) and Sigmaplot 12.5 (Systat Software, Inc., Richmond, CA, USA) software.

## 3 Results

### 3.1 Effects of H_2_O_2_ priming on summer maize yield under waterlogging stress at the V3 stage

#### 3.1.1 Yield

The summer maize yield was significantly reduced after waterlogging, but H_2_O_2_ priming could increase the yield of waterlogged summed maize (**Table 2**). In 2020, the grain yield of summer maize in C-W and H-W decreased by 24.3% and 12.4% compared with C-CK (Control treatment) respectively, C-W was 15.7% lower than H-W. The two-year results were basically consistent. The increase in grain number per ear was responsible for the significantly greater yields obtained from H_2_O_2_ priming waterlogged plants (**Table 2**). In 2020, the grain number per ear of H-W was 12.4% higher than C-W, C-W and H-W decreased by 16.5% and 6.3% compared with C-CK. The two-year results were basically consistent.

**Table 2:** Effects of H_2_O_2_ seed soaking on yield of summer maize under waterlogging stress at the seedling stage.

Year	Treatment	Ear number (ears ha^-1^)	Grains per ear	1000-kernel weight (g)	Yield (kg ha^-1^)
2020	C-CK	63125	562a	402a	14269a
	C-W	62500	469c	369b	10805c
	H-CK	62500	582a	406a	14752a
	H-W	64375	527b	372b	12505b
2021	C-CK	67500	527a	363a	12914b
	C-W	63125	437c	321c	8861d
	H-CK	67500	535a	364a	13142a
	H-W	65625	466b	349b	10682c

Values Followed by a different small letter within a column are significantly different at the 0.05 probability level. Differences between treatments were calculated within the hybrids. The same as below.

#### 3.1.2 Dry matter weight

**Figure 1:**
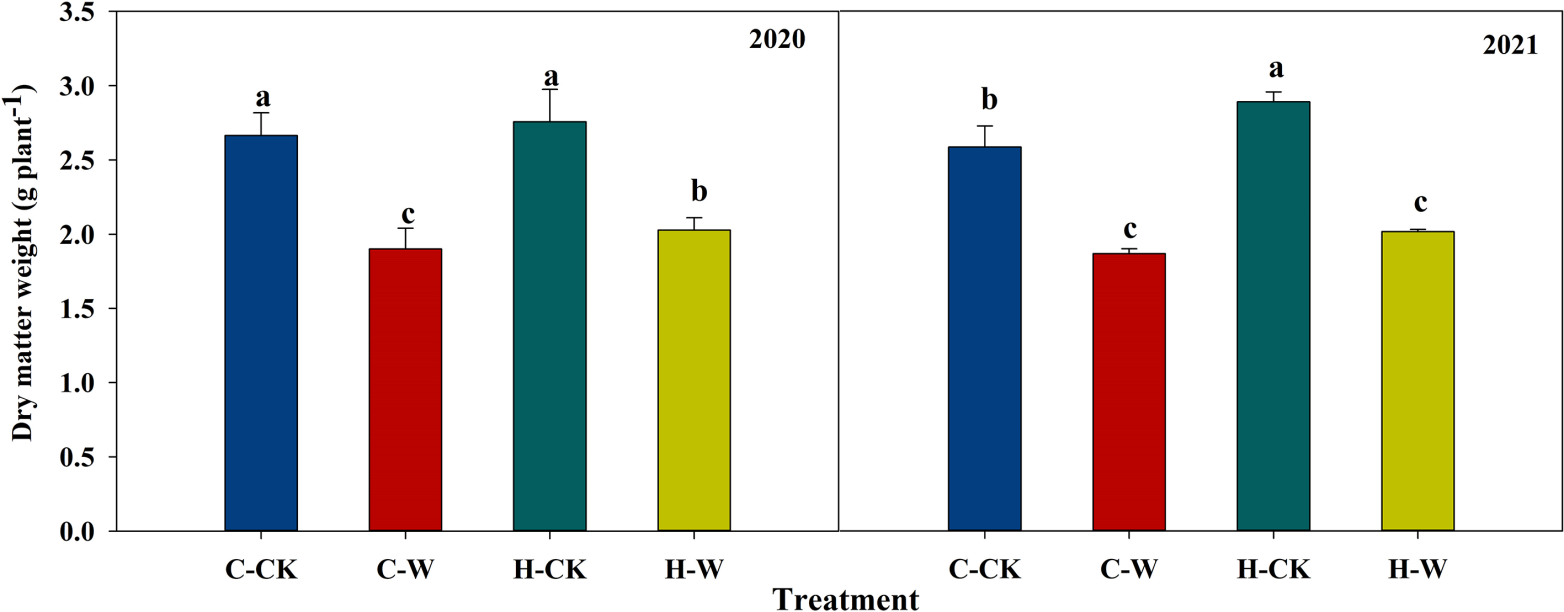
Effects of H_2_O_2_ priming on dry matter accumulation of summer maize under waterlogging stress at the seedling stage. *Note:* C-CK: Control treatment, C-W: Waterlogging for 6 days at the V3 stage; H-CK: H_2_O_2_-priming + non-waterlogging; H-W: H_2_O_2_-priming + waterlogging for 6 days at the V3 stage. The same as below.

Waterlogging reduced the dry matter accumulation of summer maize, while H_2_O_2_ priming could significantly alleviate waterlogging damages on the weight of dry matter. In 2020, compared with C-CK, waterlogging significantly decreased dry matter weight by respectively 28.7% and 23.9% on C-W and H-W respectively, and H-W was 6.7% higher than C-W. The two-year results were basically consistent (**Figure 1**).

#### 3.2 Stomatal features of maize leaves

**Figure 2:**
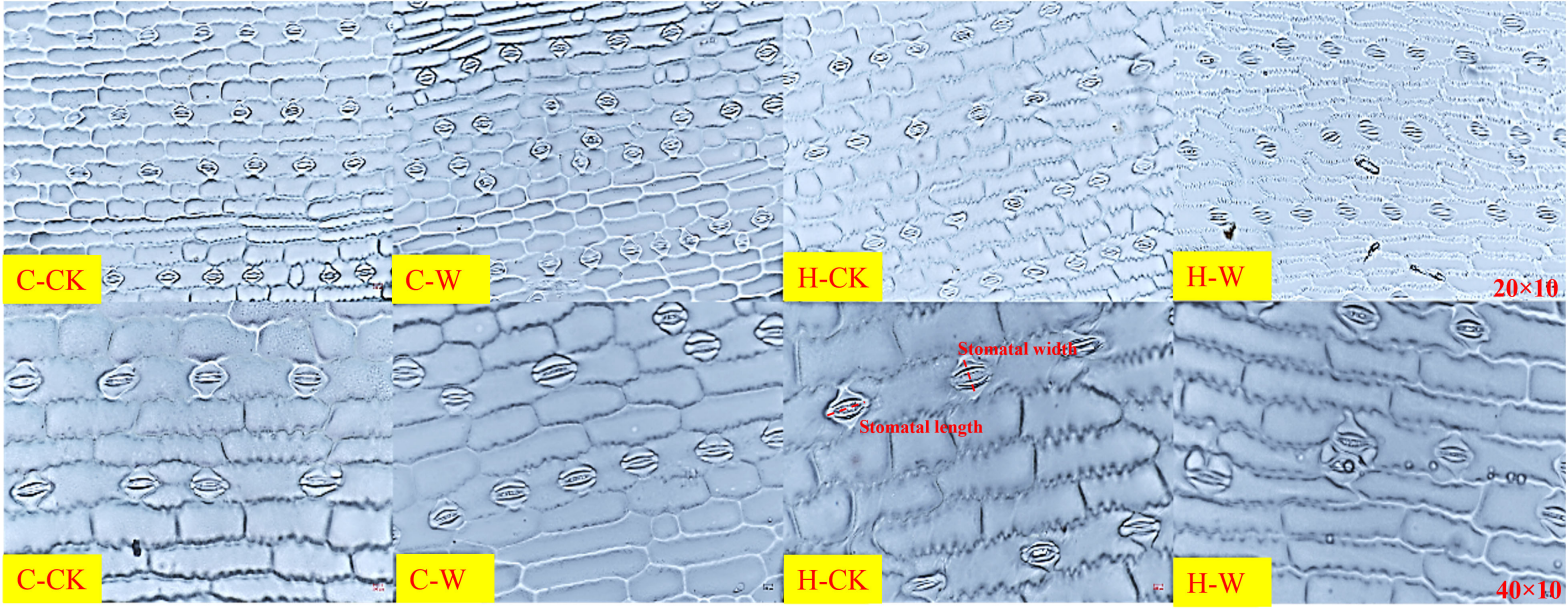
Effects of H_2_O_2_ priming on stomatal morphological characteristics of summer maize under waterlogging stress at the seedling stage.

Stomatal density, stomatal length-width ratio, and stomatal shape index of summer maize increased significantly after waterlogging, while the proportion of opening stomas, stomatal length, and width was significantly decreased. H_2_O_2_ priming alleviated the effect on the stomatal performance of waterlogged summer maize (**Table 3**). In 2020, the stomatal density (SD) of C-W was increased by 44.3% compared with C-CK, H-W decreased by 11.0% compared with C-CK, and H-W were decreased by 36.4% compared with C-W, but there was no significant difference between H-CK and C-CK. The stomatal length-width ratio (SL/SW) of H-W were decreased by 22.9% compared with C-W. The stomatal opening ratio (POS) of H-W was increased by 137.1% compared with the C-W. The two-year results were basically consistent. Additionally, the stomatal arrangement pattern was irregular, and stomatal density and the number of opening stomas were harmed after waterlogging stress at the V3 stage. H_2_O_2_ priming could significantly alleviate waterlogging damages on the stomatal arrangement pattern, density, and size of summer maize. It could be seen that the stomatal size and the degree of the stomatal opening of C-W were significantly smaller, and the open stomas were slender and flat, than those of C-CK. After priming with H_2_O_2_, the size and the degree of stomatal opening were significantly increased (**Figure 2**). The two-year results were basically consistent.

**Table 3:** Effects of H_2_O_2_ priming on leaf stomatal morphology of summer maize under waterlogging stress at the seedling stage.

Year	Treatment	SD (No mm^-2^)	SL (μm)	SW (μm)	SL/SW	SP (μm)	SA (μm)	POS (%)
2020	C-CK	28.75b	37.22b	22.74bc	1.65c	99.87c	692.40c	86.03a
	C-W	41.50a	31.21c	20.58c	1.53c	85.24d	510.17d	32.24c
	H-CK	28.20b	49.46a	23.81ab	2.08a	127.27a	968.64a	86.54a
	H-W	26.40b	48.37a	25.69a	1.88b	118.12b	892.98b	76.43b
2021	C-CK	23.20b	37.33bc	25.59ab	1.48b	105.86bc	812.32b	91.63a
	C-W	34.20a	34.25c	23.71c	1.46b	101.67c	678.32c	39.69c
	H-CK	26.40b	45.72a	26.17ab	1.75a	117.95a	934.54a	93.93a
	H-W	32.40a	39.70b	26.45a	1.50b	108.74b	825.34b	74.35b

### 3.3 Photosynthetic characteristics

#### 3.3.1 Leaf area index

**Figure 3:**
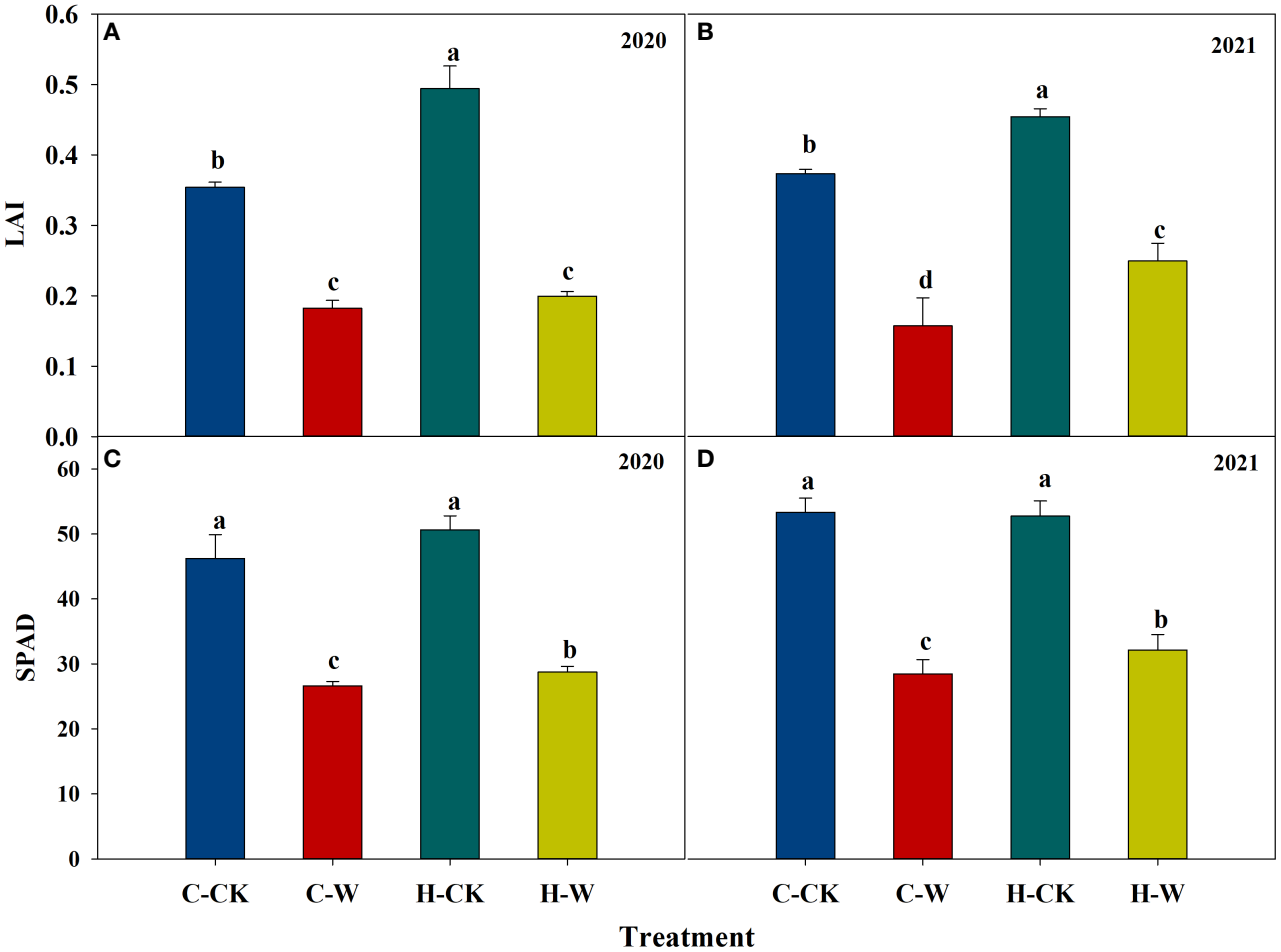
Leaf area index and Soil and plant analyzer development (SPAD) value. **(A, B)** Leaf area index in 2020 and 2021; **(C, D)** Soil and plant analyzer development (SPAD) value in 2020 and 2021.

The leaf area index (LAI) was decreased significantly after waterlogging, but H_2_O_2_ priming could alleviate the negative effects on LAI after waterlogging. In 2020, the LAI of H-W was increased by 9.4% compared with C-W, the C-W and H-W was decreased by 48.6% and 43.7% than C-CK. The two-year results were basically consistent (**Figures 3A, B**).

#### 3.3.2 Soil and plant analyzer development value

Soil and Plant Analyzer Development (SPAD) value was decreased significantly after waterlogging, but H_2_O_2_ priming could significantly alleviate the effects of waterlogging on SPAD. In 2020, pretreatment with H_2_O_2_ significantly increased the SPAD (around 8.0%) compared with C-W. The two-year results were basically consistent (**Figures 3C, D**).

#### 3.3.3 Leaf gas exchange parameters

**Figure 4:**
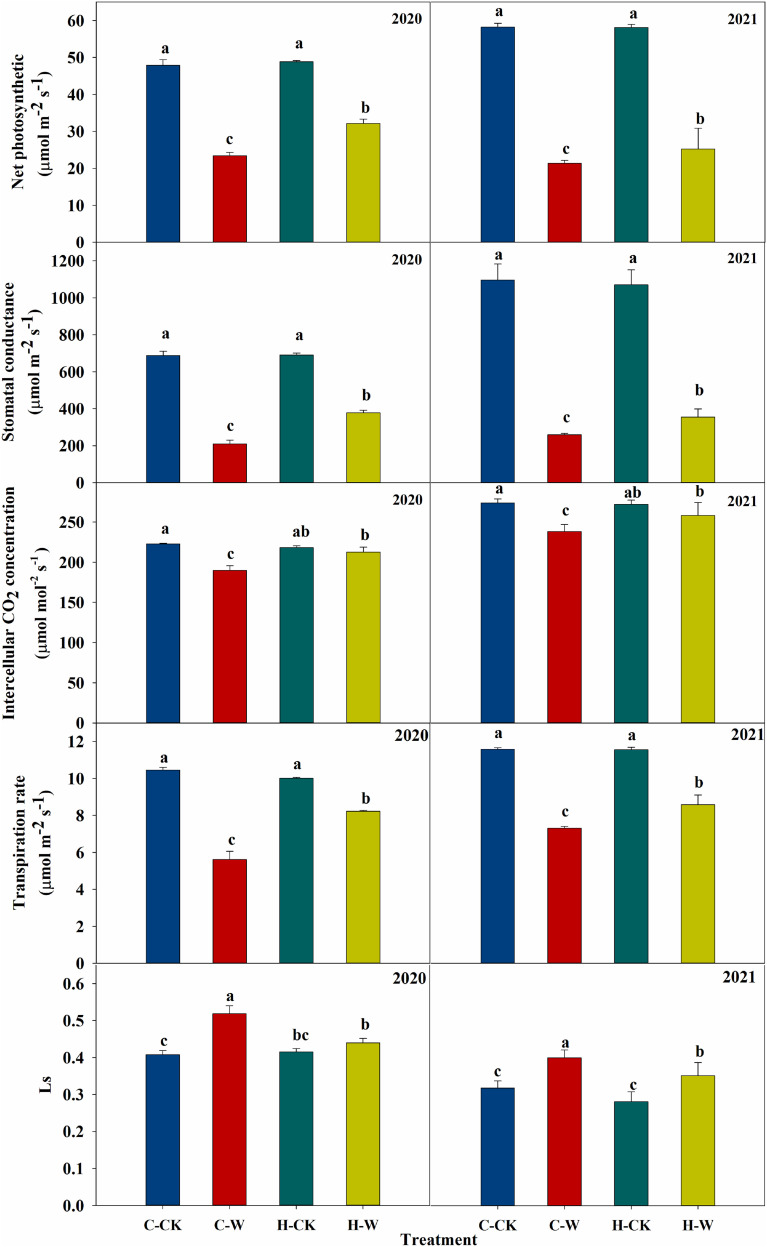
Effects of H_2_O_2_ priming on gas exchange parameters of summer maize under waterlogging stress at the seedling stage.

Leaf Pn, Gs, Ci, and Tr were significantly decreased after waterlogging. In 2020, a reduction in Pn (around 51.1%), Gs (around 69.5%), Ci (around 14.8%), and Tr (around 46.3%) was observed in C-W treatment compared with C-CK. Pretreatment with H_2_O_2_ increased the Pn (around 37.5%), Gs (around 80.4%), Ci (around 12.1%), and Tr (around 46.3%) of summer maize compared with C-W. The two-year results were basically consistent (**Figure 4**). The stomatal limitation (Ls) of summer maize increased significantly after waterlogging stress, and H_2_O_2_ priming could significantly reduce the Ls of waterlogged maize. In 2020, the stomatal limitation of H-W decreased by 15.7% compared with C-W. The two-year results were basically consistent (**Figure 4**).

### 3.4 Chlorophyll fluorescence parameters

#### 3.4.1 Changes in PSII donor/acceptor sides

**Figure 5:**
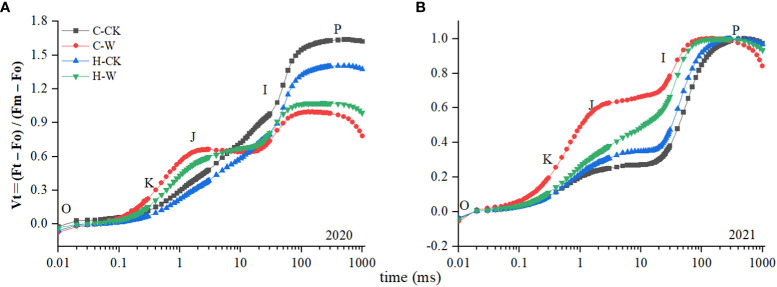
Changes in PS II donor/acceptor sides. **(A, B)** Vt curves in 2020 and 2021.

The shape of the Vt curve was obviously changed after waterlogging stress. As was shown in (**Figure 5**), the K and J step were significantly increased after waterlogging. The Wk of H-W was decreased by 14.6% compared with that of C-W. The Vj of H-W was 25.7% lower than that of C-W. The two-year results were basically consistent (**Figure 5**).

#### 3.4.2 PSII biophysical parameters derived by the JIP-test equations

##### 3.4.2.1. Phenomenological energy fluxes (per exciting cross-section - CS)

**Figure 6:**
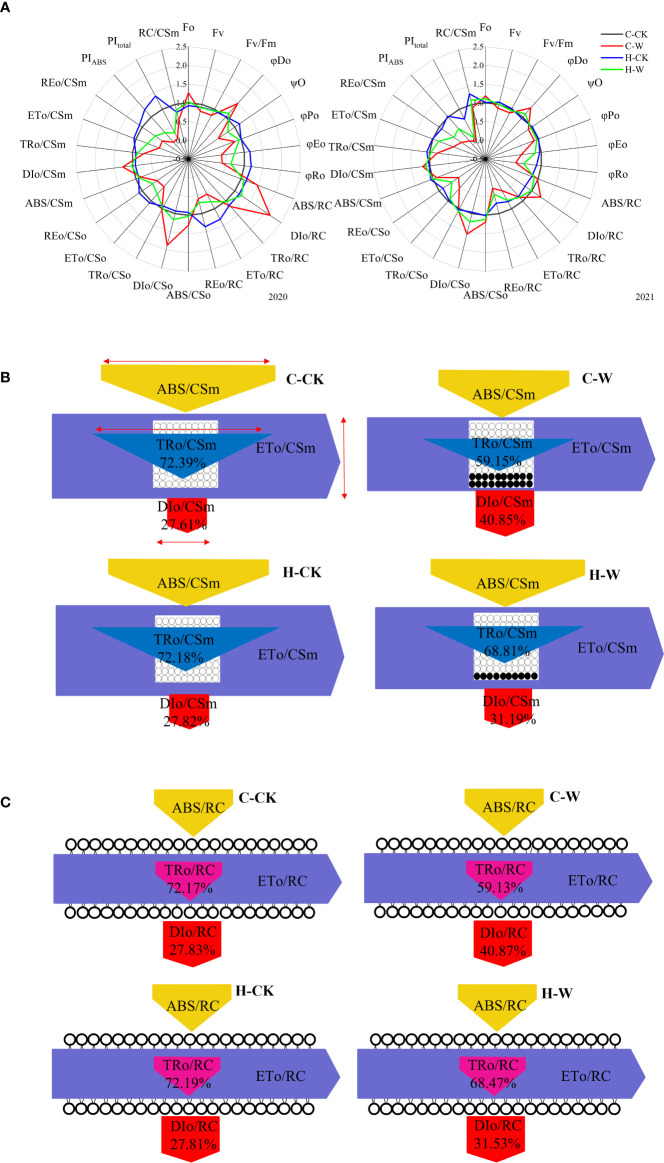
PSII biophysical parameters derived by the JIP-test equations. **(A)** Changes of JIP parameter radar images in 2020 and 2021. *Note:* The values were calculated relative to the C-CK, taken as 1, and the fluorescence parameters of other treatments were transformed into the percentage of C-CK fluorescence parameters. **(B)** Energy pipeline models of phenomenological fluxes (per cross-section, CSm) in 2020. The decrease in absorption (yellow), trapping (cyan), and electron transport (purple), can be seen as changes in the width of each arrow (red arrow = scale). The active reaction center is represented by a white circle, and the inactive reaction center is represented by a black circle. **(C)** Specific energy flux (membrane model) in 2020.

Under waterlogging stress, electron transport flux per excited CS (ETo/CSo) in maize leaves was significantly decreased, but the heat dissipation per unit area (DIo/CSo) was significantly increased. H_2_O_2_ priming significantly increased ETo/CSo and decreased DIo/CSo (**Figure 6A**). In 2020, ETo/CSo of H-W was increased by 55.3% compared with that of C-W. DIo/CSo of H-W was decreased by 37.6% compared with that of C-W. The two-year results were basically consistent (**Figure 6A**). There was a decrease in trapping flux per excited CS (TRo/CSm) (blue arrow), electron transport flux per excited CS (ETo/CSm) (purple arrow), absorption flux per excited CS (ABS/CSm) (yellow arrow), but an increase in dissipated energy flux per excited CS (DIo/CSm) (red arrow) in waterlogged maize (**Figures 6A, B**). In 2020, ETo/CSm of H-W was increased 102.7% compared with that of C-W; DIo/CSm of H-W was decreased 18.7% compared with that of C-W. The two-year results were basically consistent (**Figures 6A, B**).

##### 3.4.2.2 Specific energy fluxes (per Q_A_ reducing PSII reaction center – RC)

Waterlogging stress significantly reduced electron transport flux per RC (ETo/RC) and increased dissipated energy flux per RC (DIo/RC) in maize seedling leaves, while H_2_O_2_ priming significantly increased ETo/RC and decreased DIo/RC (**Figures 6A, C**). In 2020, RC/CSm of C-W and H-W were decreased by 42.6% and 15.6% compared with that of C-CK, respectively, and H-W was increased by 47.0% compared with that of C-W; ETo/RC of H-W was increased by 41.0% compared with that of C-W; DIo/RC of H-W was decreased by 43.1% compared with that of C-W (**Figures 6A, C**). The two-year results were basically consistent (**Figures 6A, C**).

##### 3.4.2.3 Quantum yields and efficiencies/probabilities

The probability (at t = 0) that a trapped exciton moves an electron into the electron transport chain beyond Q_A_^-^ (Ψo), the quantum yield of electron transport (φEo), and the maximum quantum yield of primary photochemistry (φPo) were decreased significantly and the quantum ratio (φDo) was increased significantly after waterlogging, while H_2_O_2_ priming significantly increased Ψo, φEo, φPo and significantly decreased φDo (**Figure 6A**). In 2020, φEo of H-W was increased by 89.9% compared with that of C-W; φPo of H-W was increased 16.2% compared with that of C-W; φDo of H-W was decreased by 23.5% compared with that of C-W (**Figure 6A**). In 2020, PI_ABS_ of H-W was increased by 352.8% compared with that of C-W (**Figure 6A**).

### 3.5 Correlation analysis

**Figure 7:**
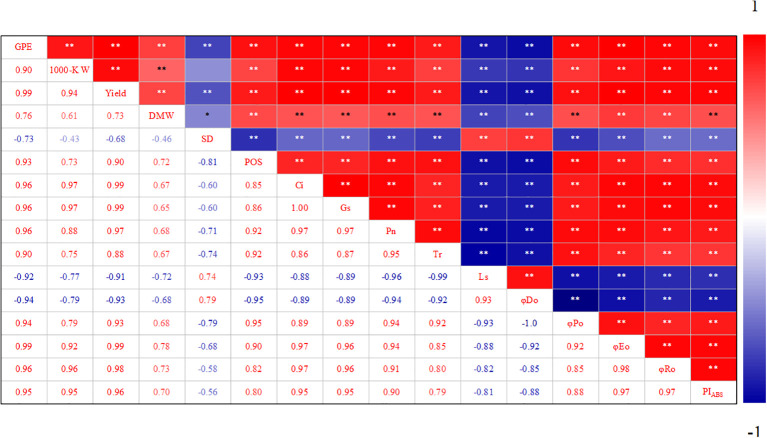
Correlation analysis of photosynthetic characteristics of soaking seeds with H_2_O_2_ under waterlogging stress. GPE: Grains per ear; 1000-K W: 1000-kernel weight; DMW: Dry matter weight; SD: Stomatal density; POS: Proportion of open stomata; Ci: Intercellular CO_2_ concentration; Gs: Stomatal conductance; Pn: The net photosynthetic rate; Tr: Transpiration rate; Ls: Calculate the pore limit value; φDo: Quantum yield of electron transport (at t = 0); φPo: The maximum quantum yield of primary photochemistry (at t = 0); φEo: Quantum yield of electron transport (at t = 0); φRo: Quantum yield for reduction of end electron acceptors at the PSI acceptor side (RE) (at t = 0); PI_ABS_: The performance index for energy conservation from exciton to the reduction of intersystem electron acceptors.

Correlation analysis showed that the net photosynthetic rate of summer maize was related to stomatal factors and non-stomatal factors. Pn was positively correlated with POS, Gs, and Ci. From the perspective of chlorophyll fluorescence, Pn was significantly positively correlated with φPo, φEo, φRo, and PI_ABS_, but negatively correlated with φDo (**Figure 7**). The decrease in grain yield of waterlogged summer maize was significantly correlated with the decreases of 1000-grain weight and grain number per spike. From the perspective of stomata, grain number per ear, 1000-grain weight, and yield were significantly negatively correlated with stomatal density (SD), but positively correlated with the proportion of open stomata (POS). Grain number per ear, 1000-grain weight, and yield was significantly positively correlated with Gs, Ci, Tr and Pn. From the perspective of chlorophyll fluorescence, grain number per ear, 1000-grain weight, and yield were significantly positively correlated with φPo, φEo, φRo, and PI_ABS_, but significantly negatively correlated with φDo (**Figure 7**).

## 4 Discussion

### 4.1 The mitigation effect of H_2_O_2_ priming on photosynthetic characteristics of waterlogged summer maize at the seedling stage

#### 4.1.1 H_2_O_2_ priming improved the stomatal performance improving photosynthetic characteristics

**Figure 8:**
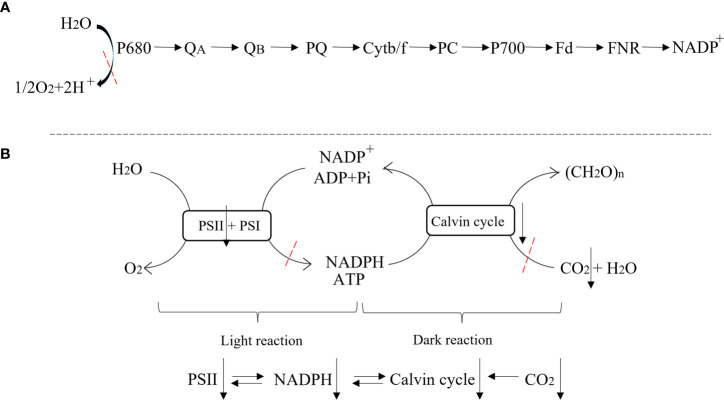
Schematic diagram of photosynthesis process. **(A)** Photosynthetic electron transport process; **(B)** Light and dark reaction.

The leaves photosynthetic characteristics are closely related to the leaf structures. The stomatal conductance and photosynthetic rate were affected by the stomata, one of the important leaf structures. The stomatal conductance was positively correlated with stomatal size and negatively correlated with stomatal density (Wu et al., 2014; Haworth et al., 2015). To better adapt to waterlogging stress, stomata would become smaller in size and larger in number which might be related to the decrease of leaf area (Xu, 1997; Yu et al., 2003; Wang et al., 2010; Jurczyk et al., 2016; Ploschuk et al., 2018; Hu et al., 2019). In this study, the stomatal length, width, and the proportion of open stomata of waterlogged summer maize leaves were markedly decreased. Additionally, the stomas of plant leaves altered from regular arrangement to random distribution in waterlogged plants, thereby enhancing the CO_2_ diffusion distance from stomas to photosynthetic parts. Accordingly, Gs and Ci of C-W were considerably decreased, while Ls were significantly increased, demonstrating that the decrease of Pn was predominantly caused by stomatal limitation factors. These results suggested that waterlogging had a grave effect on stomach traits of plants which blocked the uptake of CO_2_, and here impeding the photosynthesis, and reducing the dry matter accumulation eventually. It has been demonstrated that H_2_O_2_ priming was conducive to regulate stomatal characteristics, thus improving the gas exchange characteristics of leaves under waterlogging conditions (Castro-Duque et al., 2020). This study showed that the stomatal density of leaves reduced considerably and the stomatal size increased significantly in H_2_O_2_ priming/waterlogging-stress plants. Additionally, the proportion of opening stomata was significantly increased, and the stomata were arranged in H-W, thus enhancing the uptake of CO_2_. As a result, the Gs, Ci were increased by H_2_O_2_ priming, leading to a higher photosynthetic rate (**Figure 8**).

#### 4.1.2 H_2_O_2_ priming improved the light capture and conversion improving photosynthetic

Chlorophyll fluorescence induction kinetics is a sensitive probe to study the absorption, transformation, transmission, and distribution of sunlight energy in plants. The changes in chlorophyll fluorescence parameters can effectively reflect the characteristics of light reaction in photosynthesis (Strasserf and Srivastava, 1994; Yang et al., 2004; Kalaji et al., 2014; Li et al., 2020). PSII reaction centers were damaged and the number of active reaction centers was decreased after waterlogging (Li et al., 2005). The PSII reaction center converted the captured light energy into excitation energy. Most of the excitation energy was used to promote carbon assimilation, and the excess excitation energy was lost in the form of heat dissipation (Zuo et al., 2019). The number of RC/CSm was decreased after waterlogging, which damaged the PSII reaction centers and the antenna pigment structure. At the same time, Ro/CSm and ETo/CSm were decreased, but the DIo/CSm was increased, thus, the utilization rate of light energy per cross area, the share of energy devoted to electron transport were reduced, and heat dissipation was increased (Li et al., 2013; Liu et al., 2016). A similar conclusion could be drawn from quantum yields. Thus, waterlogging stress by decreasing the utilization of light energy and increased heat dissipation to limit the PSII photosynthetic electron transport chain. The level of electron transport inhibition at the donor or acceptor was closely related to PSII apparent activation energies (Chen et al., 2013; Sunil et al., 2020; Xing et al., 2021). This research showed that waterlogging stress significantly enhanced the K-step of summer maize, implying that the oxygen release complex (OEC) was damaged, the existence time of strong oxidant P680^+^ was prolonged, and the D_1_ protein in the reaction center of PSII was destroyed by oxidation, so as to damage the photosynthetic organs and decrease the photosynthetic activity. Additionally, the J-point was also significantly raised by waterlogging stress, indicating that the electron transfer from Q_A_ to Q_B_ was also significantly impeded, and reduced Q_A_ was accumulated, resulting in the singlet oxygen ^1^O_2_ formation and biofilm lipid peroxidation being increased.

Studies have shown that exogenous H_2_O_2_ could be used as a signal molecule to alleviate the effects of waterlogging on plants by regulating PSII efficiency and protecting chloroplasts (Castro-Duque et al., 2020; Hasanuzzaman et al., 2020). H_2_O_2_ priming significantly increased the RC/CSm, indicating that H_2_O_2_ priming protected the PSII reaction center. At the same time, H_2_O_2_ priming enhanced the electron transfer at the PSII reaction center and the donor/acceptor side of maize, decreasing the waterlogging induced raise of K and J steps, which effectively promoted the photosynthetic electron transport chain, reduced the oxidant P680^+^ and ^1^O_2_, protected the photosystem from oxidative damages and reduced the damage of membrane lipid peroxidation. Additionally, H_2_O_2_ priming significantly reduced DIo/CSm, indicating that the absorption and transfer of PSII light energy were improved. Accordingly, H_2_O_2_ priming increased the PI_ABS_, and the photochemical properties were improved, which alleviated the electron transport coupled photosynthetic phosphorylation process, and boosted the Calvin cycle. In summary, H_2_O_2_ priming effectively improved the capture, conversion, and transmission of light energy, protecting plants from photoinhibition and enhancing the carbon assimilation processes (**Figure 8**).

### 4.2 Yield

**Figure 9:**
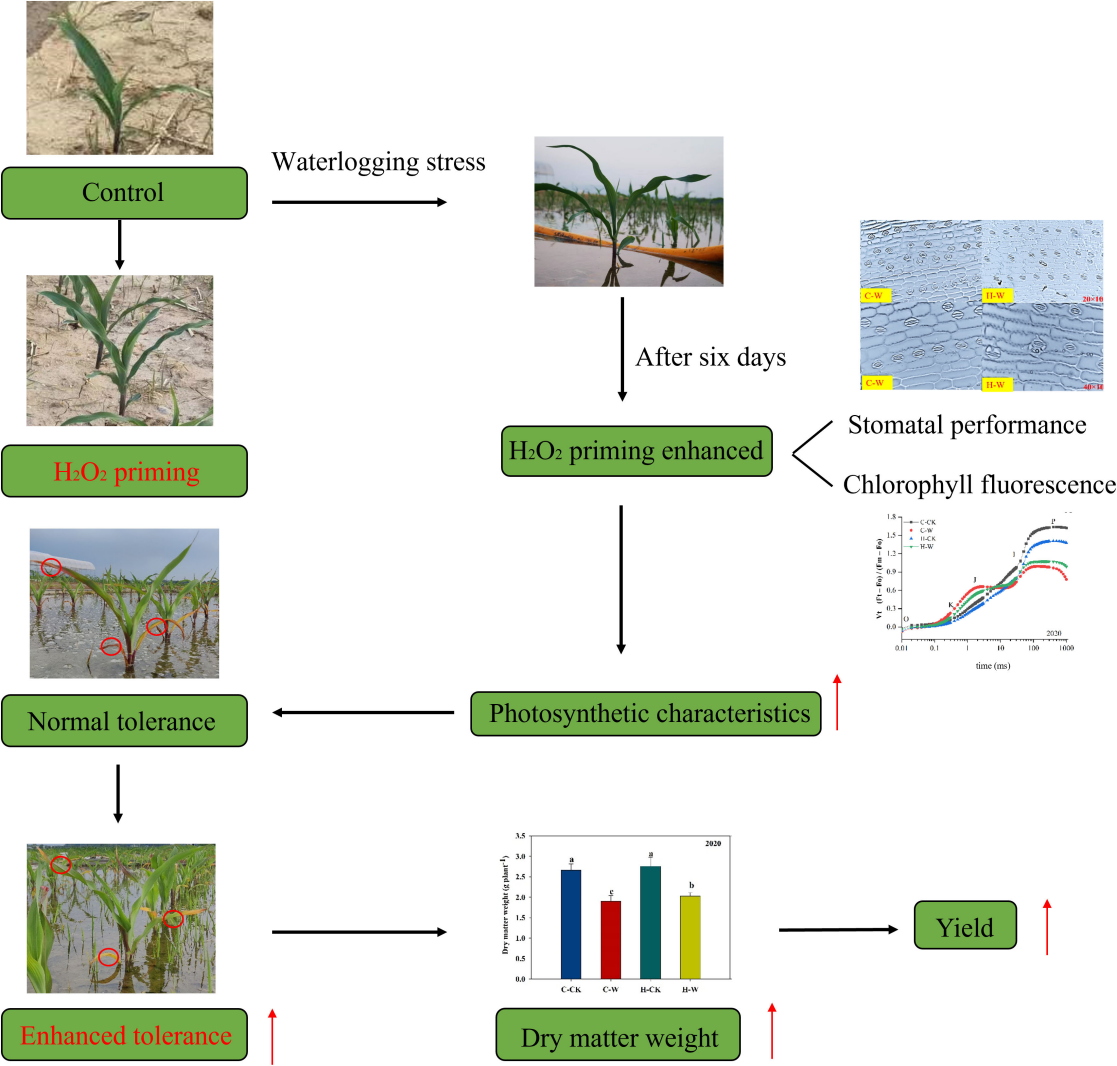
The mechanism of H_2_O_2_ priming could enhance the photosynthetic characteristics of summer maize. *Note:* Red circles indicated H_2_O_2_ priming versus flooded maize leaves, and red arrows indicated that H_2_O_2_ priming alleviates flooding stress.

The decrease in grain yield was significantly associated with the decrease in 1000-grain weight and grain number ear (Ren et al., 2016). The accumulation of photosynthetic substances depends on the strength of photosynthesis, and the formation of yield was determined by the accumulation and transport of photosynthetic substances (Ren et al., 2015). The results of this study showed that waterlogging stress could significantly reduce the yield of summer maize, and its 1000-grain weight and grain number per ear were significantly affected, with grain number per ear being the most affected. Dry matter accumulation was blocked after waterlogging, resulting in insufficient assimilation for grain development, thus leading to a reduction in grain yield (Ren et al., 2014). In summary, this study showed that waterlogging stress significantly reduced photosynthetic rate through damaging stomatal performance, PSII photoinhibition, and the light capture and conversion, and the LAI and SPAD were decreased, resulting in a blockage in dry matter accumulation, and the yield was decreased finally. However, H_2_O_2_ priming could significantly mitigate the waterlogging damages on stomatal characteristic and photosystems, protecting chloroplasts and other photosynthetic organs from oxidative damage to increase the photosynthetic rate, and increase the biomass accumulation rate and grain yield of waterlogged summer maize (**Figures 8**, **9**).

## 5 Conclusions

Waterlogging stress inhibited the light and dark response of the leaves. Waterlogging decreased Pn through stomatal limitation of CO_2_ supply and reduction of PSII photochemical efficiency by limiting the donor and acceptor side of PSII and the activity of the reaction center, which would lead to the decrease of dry matter weight and grain yield. H_2_O_2_ priming could alleviate the negative effects of on waterlogged summer maize by increasing the CO_2_ supply for carbon fixation, protecting PSII reaction centers, and enhancing electron transport to promote energy supply for carbon fixation. As a result, the photosynthetic capacity of waterlogged summer maize was significantly increased by H_2_O_2_ priming, dry matter weight and grain yield were increased eventually. In summary, H_2_O_2_ priming could modulate the waterlogging stress tolerance of summer maize: insights from photosynthetic.

## Data availability statement

The original contributions presented in the study are included in the article/**Supplementary Material**. Further inquiries can be directed to the corresponding author.

## Author contributions

SW: Conceptualization, Formal analysis, Investigation, Methodology, Writing – original draft.; JH: Conceptualization, Investigation, Methodology, Writing - Review and Editing; BR: Investigation, Resources, Writing – review and editing; BZ: Investigation, Resources, Writing – review and editing; PL: Investigation, Resources, Writing – review and editing; JZ: Conceptualization, Funding acquisition, Data curation, Supervision, Writing – review and editing. All authors contributed to the article and approved the submitted version.

## Funding

This work was supported by the Shandong Province key research and development plan (2021LZGC014-2), National Natural Science Foundation of China (32172115), the earmarked fund for CARS (CARS-02-21) and the Shandong Agricultural Application Technology Innovation Project (Grant numbers SD2019ZZ013).

## Acknowledgments

We acknowledge the support of the State Key Laboratory of Crop Biology and College of Agronomy, Shandong Agricultural University.

## Conflict of interest

The authors declare that the research was conducted in the absence of any commercial or financial relationships that could be construed as a potential conflict of interest.

## Publisher’s note

All claims expressed in this article are solely those of the authors and do not necessarily represent those of their affiliated organizations, or those of the publisher, the editors and the reviewers. Any product that may be evaluated in this article, or claim that may be made by its manufacturer, is not guaranteed or endorsed by the publisher.

## Supplementary material

Supplementary Figure 1 The details of meteorological data during maize waterlogging periods.

Supplementary Table 1 Jip-test specifies the parameter term.
