# Supplementary figures and images for: Effects of hydrogen peroxide priming on yield, photosynthetic capacity and chlorophyll fluorescence of waterlogged summer maize
Source: Front Plant Sci. 2022 Oct 21;13:1042920. doi: 10.3389/fpls.2022.1042920 (PMC9635342; doi:10.3389/fpls.2022.1042920)

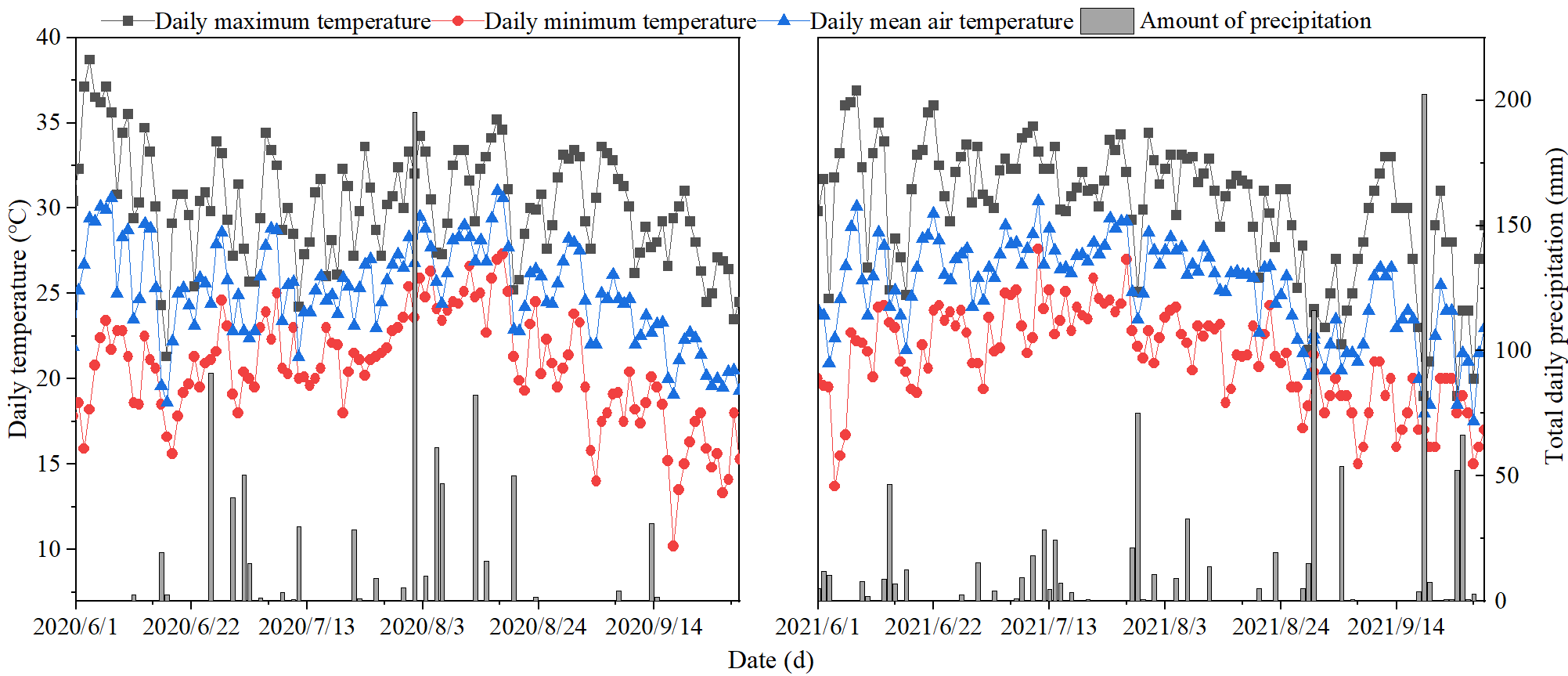

Supplement: Supplementary Figure 1 — The details of meteorological data during maize waterlogging periods. [file Image_1.tif]
