# Supplementary material for: Effects of hydrogen peroxide priming on yield, photosynthetic capacity and chlorophyll fluorescence of waterlogged summer maize
Source: Front Plant Sci. 2022 Oct 21;13:1042920. doi: 10.3389/fpls.2022.1042920 (PMC9635342; doi:10.3389/fpls.2022.1042920)
Supplement: Supplementary Table 1 — Jip-test specifies the parameter term. [file Table_1.docx]

**Supplementary Table 1 |** Jip-test specifies the parameter term.

| **Parameters** | **Biological Significances** |
| --- | --- |
| Fo | Minimal fluorescence intensity |
| Fm | Maximal fluorescence intensity |
| Fv/Fm | The ratio of maximal variable fluorescence intensity to Maximal fluorescence intensity |
| Wk | Relative variable fluorescence Fk to the amplitude Fj - Fo |
| Vj | Relative variable fluorescence at the J-step |
| Ψo | Probability (at t = 0) that a trapped exciton moves an electron into the electron transport chain beyond Q_A_^-^ |
| φEo | Quantum yield of electron transport (at t = 0) |
| φDo | Quantum yield of electron transport (at t = 0) |
| φPo | The maximum quantum yield of primary photochemistry (at t = 0) |
| φRo | Quantum yield for reduction of end electron acceptors at the PSI acceptor side (RE) (at t = 0) |
| ABS/RC | Absorption flux per RC |
| DIo/RC | Dissipated energy flux per RC (at t = 0) |
| TRo/RC | Trapped energy flux per RC (at t = 0) |
| ETo/RC | Electron transport flux per RC (at t = 0) |
| REo/RC | Electron flux reducing end electron acceptors at the PSI acceptor side, per RC (at t = 0) |
| RC/CSm | Density of active reaction centers per CS (at t = t_FM_) |
| ABS/CSo | Absorption flux per excited CS (at t = 0) |
| DIo/CSo | Dissipated energy flux per excited CS (at t = 0) |
| TRo/CSo | Trapping flux per excited CS (at t = 0) |
| ETo/CSo | Electron transport flux per excited CS (at t = 0) |
| REo/CSo | Electron flux reducing end electron acceptors at the PSI acceptor side, per CS (at t = 0) |
| ABS/CSm | Absorption flux per excited CS (at t = t_FM_) |
| TRo/CSm | Trapping flux per excited CS (at t = t_FM_) |
| ETo/CSm | Electron transport flux per excited CS (at t = t_FM_) |
| DIo/CSm | Dissipated energy flux per excited CS (at t = t_FM_) |
| REo/CSm | Electron flux reducing end electron acceptors at the PSI acceptor side, per CS (at t = t_FM_) |
| PI_ABS_ | The performance index for energy conservation from exciton to the reduction of intersystem electron acceptors |
| PI_total_ | Performance index (potential) for energy conservation from exciton to the reduction of PSI end acceptors |
